# Hepatobiliary adverse drug reactions during treatment with olaparib: an analysis of data from the EudraVigilance reporting system

**DOI:** 10.3389/fdsfr.2025.1736759

**Published:** 2026-01-09

**Authors:** Elena-Mirabela Velişcu, Cecilia Cagnotta, Maria Giuseppa Sullo, Antonietta Anatriello, Francesco Salvo, Cristina Scavone

**Affiliations:** 1 Eu2P Programme, University Bordeaux, Bordeaux, France; 2 Department of Experimental Medicine – University of Campania “Luigi Vanvitelli”, Naples, Italy; 3 Regional Center of Pharmacovigilance and Pharmacoepidemiology of Campania Region, Naples, Italy; 4 University Bordeaux, INSERM, BPH, U1219, Team AHeaD, Bordeaux, France; 5 CHU de Bordeaux, Pôle de Santé Publique, Service de Pharmacologie médicale, Centre Régional de Pharmacovigilance Bordeaux-DROM, Bordeaux, France; 6 Department of Life Science, Health, and Health Professions, Link Campus University, Rome, Italy

**Keywords:** adverse events, DILI cases, EudraVigilance, olaparib, safety profile

## Abstract

**Background:**

olaparib is a Poly ADP-Ribose Polymerase inhibitor approved for the treatment of BRCA-mutated tumors, and has been associated with hepatotoxicity, as highlighted by the Pharmacovigilance Risk Assessment Committee.

**Objectives and methods:**

to describe Drug-Induced Liver Injury (DILI) cases related to olaparib stratifying them by liver damage origin in “cholestatic”, “cytolytic”, “mixed”, “liver related investigation” and “not specified” cases, by analyzing data from EudraVigilance (EV) database.

**Results:**

344 Individual Case Safety Reports (ICSRs) reporting olaparib-induced liver injury cases were retrieved from the EV. They referred more frequently to female patients belonging to the age group 18–64 years. The majority of ICSRs (66.0%) reported serious Adverse events (AEs), classified as “Other Medically Important Condition”, while 23 ICSRs reported fatal AEs. Among the 344 DILI cases, 19.5% were classified as “cytolytic”, 3.5% as “cholestatic” and 1.5% as “mixed” origin. Most DILI cases were temporally associated with “liver-related investigations” (39.8%) and 15.1% were classified as “not specified”. A further 20.6% of cases involved PTs related to liver neoplasms (benign or malignant).

**Conclusion:**

Further pharmacovigilance studies are needed to evaluate the hepatic safety profile of olaparib. Investigating the pathophysiological mechanisms underlying olaparib-induced liver injury could offer clinicians valuable insights for its prevention and management.

## 1 Introduction

Germline mutations in the Breast Cancer gene-1 (BRCA1) and Breast Cancer gene-2 (BRCA2) are identified as significant risk factors for the development of solid tumors, including ovarian, breast, prostate and pancreatic cancers (Golan et al., 2019; Boussios et al., 2022). BRCA genes play a crucial role in DNA damage response because they encode proteins taking part in the repair process of double-strand DNA breaks by homologous recombination (Tobalina et al., 2021). The identification of these mutations has improved the development of target therapy based on poly-ADP ribose polymerase (PARP) inhibitors. PARP is a protein that supports damaged cells to repair themselves. Thus, PARP inhibitors, which currently include olaparib, rucaparib, niraparib and talazoparib, stop the PARP from doing its repair work in cancer cells leading to cancer cells’ death (Golan et al., 2019; Boussios et al., 2022).

Olaparib was the first PARP inhibitor to be approved in 2014 by the Food and Drug Administration (FDA) and the European Medicines Agency (EMA) for the oral treatment of BRCA-mutated advanced ovarian cancer in patients who had previously received three or more lines of chemotherapy (Kim et al., 2015). Throughout the years, the therapeutic indications of the drug were extended for the treatment of advanced epithelial ovarian, fallopian tube or primary peritoneal cancer after the first-line platinum-based chemotherapy, HER2-negative metastatic breast cancer, BRCA-mutated metastatic castration-resistant prostate cancer, pancreatic and endometrial cancer (Food and Drug Administration, 2024a; Food and Drug Administration, 2024b; Food and Drug Administration, 2024c; Fallah et al., 2024). Olaparib acts as a competitive inhibitor of NAD+ in the catalytic site of PARP1 and PARP2, which are involved in the repair process of DNA single-strand breaks (SSBs). Thus, olaparib leads to the accumulation of unrepaired SSBs and to the formation of dangerous double-strand breaks, which can be quickly repaired in healthy cells. In tumor cells, lacking in homologous recombination repair, the accumulation of unrepaired DNA damage is fatal, and the obstruction of replication forks leads to replication arrest. These multiple mechanisms explain the reason why tumor cells show sensitivity to olaparib and other PARP inhibitors (Bochum et al., 2018; Robson et al., 2017).

The results of both 5- and 7-year follow up analysis of phase III SOLO1 trial (Banerjee et al., 2021; DiSilvestro et al., 2023) and the evidence of a meta-analysis carried out by Guo et al. (2018) reported also the occurrence of neutropenia and thrombocytopenia. However, following the meeting held by the Pharmacovigilance Risk Assessment Committee (PRAC) on 31 July 2023 (European Medicines Agency, 2025), the EMA informed healthcare professionals (HCPs) about cases of hepatocellular damage and hepatitis occurring during the treatment with olaparib and provided special warnings and precautions for patients showing clinical symptoms or signs of hepatotoxicity or suspected Drug-Induced Liver Injury (DILI).

Generally, liver injury is identified as a common consequence of the administration of a variety of drugs, and in most cases, it requires the treatment discontinuation. When DILI is dose related, it is defined as intrinsic, direct or predictable, while when it is not dose-dependent, it can be defined as idiosyncratic (Scavone et al., 2020; Sow et al., 2021). Cytolytic liver damage represents a form of liver inflammation and injury characterized by the destruction of hepatocytes which is typically indicated by an increase in liver enzymes of five times the upper limit of normal, appearing within 90 days of drug initiation (Aithal et al., 2011; Garcia-Cortes et al., 2020). This liver damage can result from various causes, including medications. As reported by Zhu et al. (2024), DILI can be immune-mediated, resulting from the hepatic metabolism of the culprit drugs leading to reactive metabolites binding to hepatocytes and leading to autoantibodies reacting with liver-specific antigens as well as direct toxic effect on hepatocytes. Indeed, reactive metabolites may lead to hepatic damage by different mechanisms, such as oxidative stress, inflammation, cellular death due to apoptosis or necrosis and mitochondrial membrane alteration and organ dysfunction (Björnsson and Björnsson, 2022). Moreover, in some people genetically predisposed, stress can trigger innate immune responses which cause a co-stimulation for an adaptive immune response (Luo et al., 2017). Data from clinical trials revealed that almost 4% of patients receiving olaparib experienced abnormalities in routine liver tests (Rucaparib, 2017). Abnormal liver values, such as increasing ALT concentrations, have been reported also during clinical trials with rucaparib, another PARP inhibitor. On the other hand, no cases of hepatitis with jaundice or liver failure have been described for this drug. This means that rucaparib can lead to increased blood concentration of liver enzymes, but it has not been related to significant hepatotoxicity (Niraparib, 2017). The same is true for niraparib (Yang et al., 2021).

Considering that olaparib was the first PARP inhibitor to be approved being used in clinical practice and given the relevance of hepatic AEs highlighted by the PRAC, we aimed to describe cases of olaparib-induced liver injury using data from the European spontaneous reporting system (EudraVigilance) and to stratify these cases according to the liver damage origin as cytolytic, cholestatic, mixed, not specified, and liver related investigation.

## 2 Methods

### 2.1 Data source

EudraVigilance (EV) is the European pharmacovigilance database that collects Individual Case Safety Reports (ICSRs) reporting AEs related to vaccines and drugs which are authorized within the European Economic Area (EEA). The EMA manages the database on behalf of the EU drug regulatory network. EV is publicly accessible at https://www.adrreports.eu/en/search.html.

ICSRs reporting olaparing as suspected drug collected in EV from 2014 until 25-July-2025 were considered. ICSRs were downloaded in excel files that were combined and analyzed for the purposes of this study.

In EV, AEs are coded as preferred terms (PTs) using event-related information according to the Medical Dictionary for Regulatory Activities (MedDRA). MedDRA is a hierarchical dictionary that is used to code symptoms, signs, and diagnoses medical and surgical procedures, investigations, medical/social history and therapeutic indications. The MedDRA’s structure develops on multiple levels, from the Low-Level Terms (LLT) level to the System Organ Class (SOC) level. Each level provides gradually broader categories. Each PT is linked at least with a High-Level Term (HLT), a High-Level Group Term (HLGT) and a System Organ Class (SOC) level. For example, the SOC “Hepatobiliary disorders’’ contains “hepatic and hepatobiliary disorders’’ HLGT, which include “cholestasis and jaundice” as HLT, “hepatitis cholestatic” as PT and “acute cholestatic hepatitis” as LLT.

**FIGURE 1:**
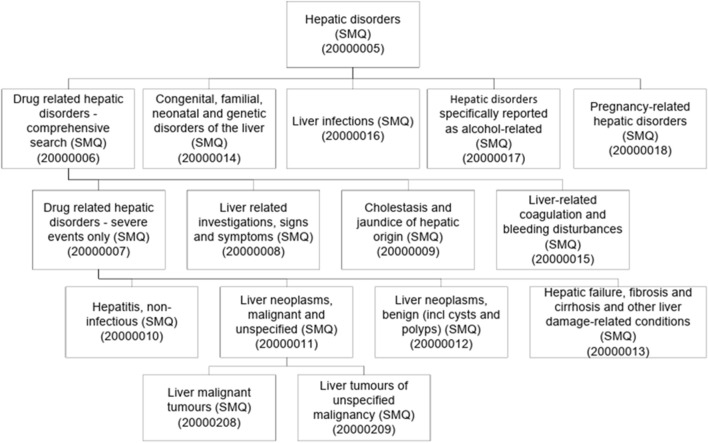
Hierarchy structure of the SMQ related to Hepatic disorders according to MedDRA. Reproduced from Introductory Guide for Standardised MedDRA Queries (SMQs), Version 25.1. © ICH. The material is used in accordance with the public copyright permission granted by ICH. No endorsement or sponsorship by ICH is implied.

In addition, Standardised MedDRA Queries (SMQs) were used to facilitate the retrieval of ICSRs related to a medical condition that can be shared by different system organs, or that can be coded using different PTs. Regarding any medical condition of interest, SMQs contain terms related to its signs, symptoms, diagnoses, syndromes, physical findings, laboratory and other physiological test data (Brown, 2003; Sakaeda et al., 2013; Mozzicato, 2007). For example, two PTs of two different SOCs, such as “hepatitis” (belonging to the SOC “Hepatobiliary disorders”) and “transaminases increased” (belonging to the SOC “Investigations”), can be grouped in the same SMQ “Hepatic disorders”. Each SMQ contains both narrow and broad search terms to describe a medical condition in various forms. Narrow terms are those that are highly likely to represent the condition of interest, while broad terms are less specific to the condition and require additional terms to qualify a case for the SMQ. In addition, some SMQs have a hierarchical structure and contain subordinate SMQs. For example, the SMQ “Liver-related coagulation and bleeding disturbances” is a sub-SMQ of Drug related hepatic disorders–comprehensive search (SMQ), which is itself a sub-SMQ of Hepatic disorder (SMQ). The SMQ on hepatic disorders includes events that can be drug-related or not. The hierarchy structure of this SMQ consists of 13 sub-SMQs (Figure 1) (Meddra, 2025).

### 2.2 Case definition

To select DILI cases related to olaparib, we considered the narrow terms of the SMQs “Drug related hepatic disorders–comprehensive search” (20000006), excluding the SMQs that do not contain PTs suggestive of drug-related liver injury (“Congenital, familial, neonatal and genetic disorders of the liver”, “Liver infections”, “Hepatic disorders specifically reported as alcohol-related”, “Pregnancy-related hepatic disorders”). We retrieved the ICSRs reporting olaparib as suspected drug and narrow PTs of the SMQ 20000006, collected in EV from 2014 until 25-July-2025, using the line-listing function. We identified potential duplicates by comparing case IDs and removed them from the dataset.

A case of DILI was defined as an ICSR reporting olaparib as suspected drug, and at least one narrow PT related to the SMQs “Drug related hepatic disorders–comprehensive search” (20000006). Moreover, cases of DILI were stratified by liver damage origin (Supplementary Table S1):

1. Cytolytic: ICSRs reporting at least one PT belonging to the SMQs “Drug related hepatic disorders–severe events only” (20000007) and suggesting hepatocyte cytolysis.
2. Cholestatic: ICSRs reporting at least one PT belonging to the SMQs “Cholestasis and jaundice of hepatic origin” (20000009) and suggesting an alteration of the normal flow of bile from the liver to the intestine.
3. Mixed: ICSRs reporting both PTs of cytolytic origin and PTs of cholestatic origin.
4. Not specified: ICSRs reporting PTs not specifically attributable to cytolytic or cholestatic origin (i.e., PTs belonging to both the SMQs “Drug related hepatic disorders–severe events only” and “Cholestasis and jaundice of hepatic origin”).
5. Liver related investigation: ICSRs reporting only PTs belonging to the sub-SMQs “Liver related investigations, signs and symptoms” (20000008) or “Liver related coagulation and bleeding disturbances” (20000015).
6. Liver neoplasm or nonspecific signs: ICSRs reporting at least one PT related to liver neoplasm (benign or malignant) or only PTs related to nonspecific signs and symptoms.

The screening of cases and the final selection of DILI cases was carried out by a team made up of pharmacists and pharmacologists, including a clinician expert in pharmacovigilance.

### 2.3 Data analyses

All retrieved ICSRs were analyzed in terms of patients’ age group and gender, primary source country for regulatory purposes (EEA or non-EEA), reporter type (HCP or non-HCP), distribution of all AEs reported in each ICSR by SOC, type of DILIs, seriousness and outcome degrees. Specifically, a case was defined as “serious’’ when it resulted in death, in persistent or significant disability or incapacity, when it caused or prolonged hospitalization, when it was life-threatening, it determined a congenital anomaly/birth defect, or it was classified under other medically important conditions (European Medicines Agency, 2024). The outcome was instead classified as “Fatal”, “Not Recovered/Not Resolved’’, “Recovered/Resolved with sequelae”, “Recovered/Resolved’’, “Recovering/Resolving’’ and “Unknown’’. The outcome with the lower level of resolution was chosen for classification whether an ICSR reported two or more PTs of interest with different outcomes (EMA, 2023). Two independent reviewers (EMV and CC) analyzed excel files and performed the process for phenotype assignment *w*th the support of the multidisciplinary team previously mentioned. Any discrepancy was resolved through discussion.2.4. Ethical standards.

Safety data extracted from the spontaneous reporting system comply with ethical standards and are anonymous. Therefore, no ethical measures were enforced further.

## 3 Results

### 3.1 Main characteristics of DILI cases

During the study period, 344 ICSRs reporting olaparib-induced liver injury cases were retrieved from the EV (Table 1). DILI cases referred more frequently to patients belonging to the age group 18–64 years (44.5%) and to the female gender (82%). The primary source country was mostly non-European Economic Area (EEA) (64.8%) and the primary source qualification was mainly represented by HCPs (84.0%). Regarding the seriousness degree, the majority of ICSRs reported AEs that were classified as “Other Medically Important Condition’’ (41%) and “Non serious” (34.0%), while the outcome was mainly reported as unknown (54.4%) and “Recovered/Resolved” (16.9%). Overall, there were 23 ICSRs related to fatal AEs (please see section 3.3 Fatal cases).

**TABLE 1:** Demographic and clinical characteristics of Drug-Induced Liver Injury (DILI) cases with olaparib as suspected drug and retrieved from the EudraVigilance databases from 2014 until 22- July −2025.

ICSRs (N = 344)	​	n (%)
Age group	18–64 Years	153 (44.5)
	65–85 Years	75 (21.8)
	More than 85 Years	2 (0.6)
	Not specified	114 (33.1)
Sex	Female	282 (82.0)
	Male	57 (16.5)
	NA	5 (1.5)
Report type	Spontaneous	344 (100.0)
Source qualification	Non-healthcare professional	55 (16.0)
	Healthcare professional	289 (84.0)
Source country	Non-european economic area	223 (64.8)
	European economic area	121 (35.2)
Seriousness	Non serious	117 (34.0)
	Other medically important condition	141 (41.0)
	Caused/Prolonged hospitalisation	52 (15.1)
	Results in death	23 (6.7)
	Life threatening	9 (2.6)
	Disabling	2 (0.6)
Outcome	Not reported	187 (54.4)
	Recovered/Resolved	58 (16.9)
	Recovering/Resolving	43 (12.5)
	Not recovered/Not resolved	33 (9.6)
	Fatal	23 (6.7)
	Recovered/Resolved with sequelae	0

NA, not available.

**FIGURE 2:**
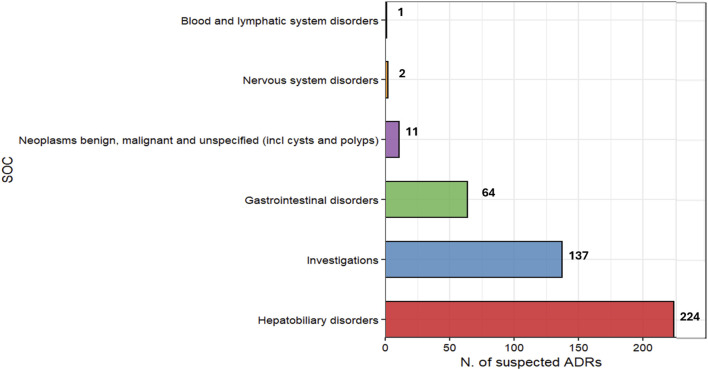
Distribution of AEs included in the Drug-Induced Liver Injury (DILI) cases reporting olaparib as suspected drug by System Organ Classes (SOCs).

Overall, the retrieved ICSRs covered 439 PTs, which were mostly related to the SOCs “Hepatobiliary disorders” (51.0%), “Investigations” (31.2%), “Gastrointestinal disorders” (14.6%), “Neoplasm benign, malignant and unspecified (incl cysts and polyps)” (2.5%), “Nervous system disorders” (0.5%), “Blood and lymphatic system disorders” (0.2%) (Figure 2).

### 3.2 Stratification of DILI cases by liver damage origin

Among the 344 DILI cases, 67 (19.5%) were classified as “cytolytic”, 12 (3.5%) as “cholestatic”, and 5 (1.5%) as “mixed” origin, as the corresponding ICSRs included PTs of both cytolytic and cholestatic type. Most DILI cases were temporally associated with “liver-related investigations” (39.8%) and 15.1% were classified as “not specified”, as the reported PTs were not specifically attributable to cytolytic or cholestatic origin. A further 20.6% of cases involved PTs related to liver neoplasms (benign or malignant) or only PTs related to non-specific signs and symptoms (Table 2).

**TABLE 2:** Demographic and clinical characteristics of Drug-Induced Liver Injury (DILI) cases by liver damage origin with olaparib as suspected drug and retrieved from the EudraVigilance databases from 2014 until 22- July −2025.

Liver damage origin	Cytolytic^a^ (19.5%)	Cholestatic^b^ (3.5%)	Mixed^c^ (1.5%)	Not specified^d^ (15.1%)	Liver related investigation^e^ (39.8%)	Liver neoplasm or nonspecific signs^f^ (20.6%)
ICSRs n (%)	67 (100)	12 (100)	5 (100)	52 (100)	137 (100)	71 (100)
Age group						
18–64 Years	26 (40.0)	7 (58.3)	3 (60.0)	28 (51.9)	62 (45.3)	27 (38.0)
65–85 Years	16 (24.6)	3 (25.0)	0 (0.0)	12 (22.2)	27 (19.7)	17 (23.9)
Not specified	0 (0.0)	0 (0.0)	0 (0.0)	0 (0.0)	2 (1.5)	0 (0.0)
More than 85 Years	23 (35.4)	2 (16.7)	2 (40.0)	14 (25.9)	46 (33.6)	27 (38.0)
Sex						
Female	52 (80.0)	8 (66.7)	5 (100.0)	44 (81.5)	108 (78.8)	65 (91.5)
Male	13 (20.0)	3 (25.0)	0 (0.0)	8 (14.8)	27 (19.7)	6 (8.5)
NA	0 (0.0)	1 (8.3)	0 (0.0)	2 (3.7)	2 (1.5)	0 (0.0)
Report_type						
Spontaneous	65 (100.0)	12 (100.0)	5 (100.0)	54 (100.0)	137 (100.0)	71 (100.0)
Source_qualification						
Non healthcare professional	8 (12.3)	2 (16.7)	0 (0.0)	4 (7.4)	12 (8.8)	29 (40.8)
Healthcare professional	57 (87.7)	10 (83.3)	5 (100.0)	50 (92.6)	125 (91.2)	42 (59.2)
Source_country						
Non european economic area	42 (64.6)	6 (50.0)	3 (60.0)	34 (63.0)	84 (61.3)	54 (76.1)
European economic area	23 (35.4)	6 (50.0)	2 (40.0)	20 (37.0)	53 (38.7)	17 (23.9)
Seriousness						
Non serious	8 (12.0)	2 (16.7)	0 (0.0)	10 (19.2)	83 (60.6)	14 (19.7)
Other medically important condition	23 (34.3)	2 (16.7)	1 (20.0)	34 (65.4)	43 (31.4)	38 (53.5)
Caused/Prolonged hospitalisation	20 (29.8)	5 (41.7)	4 (80.0)	7 (13.5)	6 (4.4)	10 (14.1)
Results in death	12 (17.9)	2 (16.7)	0 (0.0)	1 (1.9)	1 (0.7)	7 (9.9)
Life threatening	3 (4.6)	1 (8.3)	0 (0.0)	0 (0.0)	4 (2.9)	1 (1.4)
Disabling	1 (1.5)	0 (0.0)	0 (0.0)	0 (0.0)	0 (0.0)	1 (1.4)
Outcome						
Not reported	29 (43.3)	6 (50.0)	1 (20.0)	26 (50.0)	72 (52.6)	54 (76.1)
Not recovered/Not resolved	7 (10.8)	2 (16.7)	1 (20.0)	6 (11.1)	12 (8.8)	5 (7.0)
Recovered/Resolved	13 (20.0)	1 (8.3)	1 (20.0)	6 (11.1)	34 (24.8)	4 (5.6)
Recovering/Resolving	7 (10.8)	1 (8.3)	2 (40.0)	13 (24.1)	18 (13.1)	1 (1.4)
Fatal	12 (17.9)	2 (16.7)	0 (0.0)	1 (1.9)	1 (0.7)	7 (9.9)

^a^ Cytolytic: ICSRs, reporting at least one PT, belonging to the SMQs “Drug related hepatic disorders–severe events only” (20000007) and suggesting hepatocyte cytolysis.

^b^ Cholestatic: ICSRs, reporting at least one PT, belonging to the SMQs “Cholestasis and jaundice of hepatic origin” (20000009) and suggesting an alteration of the normal flow of bile from the liver to the intestine.

^c^ Mixed: ICSRs, reporting both PTs, of cytolytic origin and PTs, of cholestatic origin.

^d^ Not specified: ICSRs, reporting PTs, not specifically attributable to cytolytic or cholestatic origin (i.e., PTs, belonging to both the SMQs “Drug related hepatic disorders–severe events only” and “Cholestasis and jaundice of hepatic origin”).

^e^ Liver related investigation: ICSRs, reporting only PTs, belonging to the sub-SMQs “Liver related investigations, signs and symptoms” (20000008) or “Liver related coagulation and bleeding disturbances” (20000015).

^f^ Liver neoplasm or nonspecific signs: ICSRs, reporting at least one PT, related to liver neoplasm (benign or malignant) or only PTs, related to nonspecific signs and symptoms.

NA, not available.

Also stratifying by the origin of liver damage, DILI cases referred more frequently to patients belonging to the age group 18–64 years and to the female gender. For each category, the primary source country for regulatory purposes was mostly non-EEA and more than 50% of ICSRs were reported by HCPs. Regarding the seriousness degree, for each category the majority of ICSRs were classified as serious (88.0% for “cytolytic” cases, 83.3% for “cholestatic” cases, 100.0% for “mixed” cases, 80.8% for “not specified”cases and 80.3% for “liver neoplasm or nonspecific signs” cases). Cases classified as “liver-related investigations” were mostly reported as “Non serious” (60.6%). In addition, the outcome was mainly reported as unknown for all categories, while fatal cases were related only to cytolytic, cholestatic and liver neoplasm or nonspecific signs cases (Table 2).

In cases of cytolytic origin, the most reported PTs were hepatic failure (32.3%), hepatic cytolysis (12.3%) and hepatitis (9.2%), while for cases of cholestatic origin, jaundice (25.0%), cholestasis (25.0%) and jaundice cholestatic (25.0%) were the most reported PTs. Mixed cases, which are ICSRs reporting both PTs of cytolytic and cholestatic origins, mainly reported hepatic cytolysis, hepatitis cholestatic and jaundice as PTs. In cases of not specified origin, liver disorder, drug-induced liver injury, hepatotoxicity and liver injury prevailed. Cases temporally associated with liver investigations most frequently reported hepatic function abnormal, alanine aminotransferase increased, and hepatic enzyme increased. Among the liver neoplasm or nonspecific signs cases, the most frequent PTs were ascites, hepatic cancer, and hepatic cysts (Table 3).

**TABLE 3:** Distribution of the Preferred Terms (PTs) by DILI cases classified by liver damage origin.

Liver damage origin	PTs	
	n	(%)
**Cytolitic** ^a^	**97**	**(100)**
Hepatic failure	21	(21.6)
Hepatic cytolysis	8	(8.2)
Hepatitis	6	(6.2)
Hepatic cirrhosis	5	(5.1)
Hepatic lesion	5	(5.1)
**Cholestatic** ^b^	**18**	**(100)**
Cholestasis	3	(16.7)
Jaundice	3	(16.7)
Jaundice cholestatic	3	(16.7)
Ocular icterus	1	(5.5)
**Mixed** ^c^	**14**	**(100)**
Hepatic cytolysis	3	(21.4)
Hepatitis cholestatic	3	(21.4)
Jaundice	2	(14.3)
Acute hepatic failure	1	(7.1)
Cholestasis	1	(7.1)
**Not specified** ^d^	**61**	**(100)**
Liver disorder	21	(34.4)
Drug-induced liver injury	13	(21.3)
Hepatotoxicity	12	(19.7)
Liver injury	7	(11.5)
**Liver related investigation** ^e^	**176**	**(100)**
Hepatic function abnormal	41	(23.3)
Alanine aminotransferase increased	27	(15.3)
Hepatic enzyme increased	19	(10.8)
Aspartate aminotransferase increased	18	(10.2)
Transaminases increased	17	(9.7)
**Liver neoplasm or nonspecific signs** ^f^	**73**	**(100)**
Ascites	56	(76.7)
Hepatic cancer	4	(5.5)
Hepatic cyst	4	(5.5)
Hepatic cancer recurrent	3	(4.1)
Hepatic neoplasm	3	(4.1)

^a^ Cytolytic: ICSRs, reporting at least one PT, belonging to the SMQs “Drug related hepatic disorders–severe events only” (20000007) and suggesting hepatocyte cytolysis.

^b^ Cholestatic: ICSRs, reporting at least one PT, belonging to the SMQs “Cholestasis and jaundice of hepatic origin” (20000009) and suggesting an alteration of the normal flow of bile from the liver to the intestine.

^c^ Mixed: ICSRs, reporting both PTs, of cytolytic origin and PTs, of cholestatic origin.

^d^ Not specified: ICSRs, reporting PTs, not specifically attributable to cytolytic or cholestatic origin (i.e., PTs, belonging to both the SMQs “Drug related hepatic disorders–severe events only” and “Cholestasis and jaundice of hepatic origin”).

^e^ Liver related investigation: ICSRs, reporting only PTs, belonging to the sub-SMQs “Liver related investigations, signs and symptoms” (20000008) or “Liver related coagulation and bleeding disturbances” (20000015).

^f^ Liver neoplasm or nonspecific signs: ICSRs, reporting at least one PT, related to liver neoplasm (benign or malignant) or only PTs, related to nonspecific signs and symptoms.

Bold values indicate liver damage origin categories; the Preferred Terms (PTs) listed below are classified within these categories.

### 3.3 Fatal cases

Among the 344 DILI cases, there were 23 ICSRs related to fatal AEs, mostly referred to female gender (87.0%), as for the overall. In the majority of ICSRs reporting fatal cases, the age group was not specified (56.5%), while the remaining percentage was equally balanced between the age group 18–64 years and 65–85 years. The primary source country was mostly non-European Economic Area (EEA) (82.6%) and the primary source qualification was mainly represented by HCPs (65.2%), as for the overall DILI cases.

Most of the fatal cases, were classified as “cytolytic” (52.2%), and as “liver neoplasm or nonspecific signs” (30.4%), as for the overall DILI cases (Table 4).

**TABLE 4:** Demographic and clinical characteristics of fatal cases.

Fatal cases	Overall
ICSRs n (%)	23
Age group (%)	
18–64 Years	5 (21.7)
65–85 Years	5 (21.7)
Not specified	13 (56.5)
Sex (%)	
Female	20 (87.0)
Male	3 (13.0)
source_qualification (%)	
Healthcare professional	15 (65.2)
Non healthcare professional	8 (34.8)
source_country (%)	
European economic area	4 (17.4)
Non european economic area	19 (82.6)
Liver damage origin (%)	
Cytolytic	12 (52.2)
Liver neoplasm or nonspecific signs	7 (30.4)
Not specified	1 (4.3)
Cholestatic	2 (8.7)
Liver related investigation	1 (4.3)

^a^ Cytolytic: ICSRs, reporting at least one PT, belonging to the SMQs “Drug related hepatic disorders–severe events only” (20000007) and suggesting hepatocyte cytolysis.

^b^ Cholestatic: ICSRs, reporting at least one PT, belonging to the SMQs “Cholestasis and jaundice of hepatic origin” (20000009) and suggesting an alteration of the normal flow of bile from the liver to the intestine.

^c^ Mixed: ICSRs, reporting both PTs, of cytolytic origin and PTs, of cholestatic origin.

^d^ Not specified: ICSRs, reporting PTs, not specifically attributable to cytolytic or cholestatic origin (i.e., PTs, belonging to both the SMQs “Drug related hepatic disorders–severe events only” and “Cholestasis and jaundice of hepatic origin”).

^e^ Liver related investigation: ICSRs, reporting only PTs, belonging to the sub-SMQs “Liver related investigations, signs and symptoms” (20000008) or “Liver related coagulation and bleeding disturbances” (20000015).

^f^ Liver neoplasm or nonspecific signs: ICSRs, reporting at least one PT, related to liver neoplasm (benign or malignant) or only PTs, related to nonspecific signs and symptoms.

## 4 Discussion

Using data from the EV database, we analyzed the safety profile of olaparib in terms of hepatobiliary AEs. This decision derived from a recent question highlighted by the PRAC about the risk of hepatocellular damage associated with this drug (Kim et al., 2015).

We found 344 DILI cases, ICSRs reporting at least one narrow PT related to the SMQs “Drug related hepatic disorders–comprehensive search” and olaparib as suspected drug. Patients experiencing olaparib-induced hepatotoxicity were mostly female. The higher female frequency is expected considering that ovarian cancer represents the first therapeutic indication olaparib was approved for, followed by HER2-negative metastatic breast cancer and, only in 2022, its indication was extended for the treatment of metastatic castration-resistant prostate cancer too (Food and Drug Administration, 2024a; Food and Drug Administration, 2024b; Food and Drug Administration, 2024c; Rydberg et al., 2018). Moreover, it is well known that some gender differences between women and men exist in terms of frequency of experiencing AEs. Women, for instance, tend to experience AEs more commonly than men probably because that sex hormones play in both pharmacokinetics and pharmacodynamics of drugs (Spoletini et al., 2012). In women menstrual cycle, modifications in the concentration of sexual steroids, the use of oral contraceptives or hormonal substitutive therapy are factors which may influence drug response. On the other hand, the impact of androgens in men is not so relevant thanks to the regular levels of these hormones in blood (Scavone et al., 2020; Velişcu et al., 2023; Nicoletti et al., 2023; Web et al., 2017).

We also found that DILI cases related to olaparib occurred more commonly in patients belonging to the age group 18–64 years followed by the age group 65–85 years. This is expected considering that, although ovarian cancer can be diagnosed in women of all ages, its occurrence is quite rare among women aged <40 years considering that the risk increases both with the age and after menopause. Epithelial tumors represent more than 90% above age 40 (Doubeni et al., 2016; Rahman and Helvie, 2022). Similarly, in breast cancer pathogenesis, age represents an important risk factor for its occurrence with a median age of new cases between 40 and 49 years. This is generally the age range in which the initiation of screening mammography is recommended (Tufoni et al., 2018).

The hepatic-related AEs were mainly classified as serious (above 60%), and 23 ICSRs reported fatal cases. When stratifying DILI cases by liver damage origin, more than 50% referred to cases of cytolytic origin and liver-related investigation. On the other hand, almost 3.5% of DILI cases were classified as of cholestatic origin, \while 1.5% were classified as mixed cases.

These results are in line with some case reports recently published. The first one describes the case of a 61-year-old woman with BRCA1 mutated ovarian cystadenocarcinoma and no previous history of abnormal liver function. After starting the therapy with olaparib, she has been hospitalized for noticeable hypertransaminasemia (Alshelleh et al., 2022). Another case report describes the clinical history of a 56-year-old woman with advanced endometrioid and serous carcinoma, firstly treated with neoadjuvant chemotherapy based on carboplatin and paclitaxel. After cancer relapsed, she started retreatment with carboplatin and doxorubicin for a total of 6 cycles. Olaparib was eventually administered because of BRCA1 mutation. Three months after the initiation of olaparib, a severe acute liver injury required the hospitalization of the patient. No use of alcohol was evidenced. Physical examination showed jaundice and no hepatic encephalopathy, while mild hepatomegaly with periportal edema was evidenced by computed tomography (Zhu et al., 2024). Lastly, Zhu K et al. reported the case of a 70-year-old woman who experienced significant a severe hepatocellular injury after initiating first line maintenance olaparib. Her clinical conditions improved after the culprit drug’s discontinuation but promptly worsened upon rechallenge (Kumachev and Wu, 2021).

Most of the fatal cases retrieved by our analysis were classified as “cytolytic” and as “liver neoplasm or nonspecific signs”. In contrast with our results, we found no fatal cases of hepatotoxicity related to olaparib treatment reported in literature. This evidence could suggest that the fatality of these cases may be partially explained by the presence of hepatic neoplasm or the progression of disease.

Therefore, according to data available from the literature and the results from our study, special attention is required regarding hepatobiliary disorders for olaparib, and further studies are needed to better evaluate the safety profile of the drug. According to the PRAC recommendations, HCPs should constantly monitor the liver function tests during treatment with olaparib. Moreover, in case of potential DILI develops, proper clinical measures should be taken, such as treatment interruption. In case DILI is confirmed, the treatment can be even discontinued.

Our study has some limitations and strengths. First, we used data from the EV database that, like any other spontaneous reporting system, suffers from the well-known under-reporting phenomena as well as the poor quality and lack of information listed in each ICSR. Indeed, it is known that many ADRs are never reported by healthcare professionals or patients, especially if they are mild or expected. Similarly, ICSRs may contain missing or incomplete or inconsistent clinical information (such as, for example, data related to the outcome that in our study was missing in more than 50% of retrieved ICSRs). For instance, we could not make any analysis concerning the time to onset of olaparib-induced liver injury and similarly we could not exclude the effects deriving from concomitant diseases or medications on the occurrence of DILI. Another important limitation is not knowing whether the reported patients experiencing “liver neoplasm or non-specific signs” have had liver metastases before starting treatment with olaparib. Thus, the overall results should be interpreted with caution. In addition, as any data based on the spontaneous reporting system, our data lack of denominator data and thus we do not know how many people actually used the drug, at what dose, or for how long. Therefore, incidence rates cannot be calculated. What’s more, we could not establish causality considering that EV is designed to detect signals, not to prove that a drug causes an event and we cannot rule out that many events could be related with the illness rather than the drug (leading to a confounding by indication). Lastly, since we classified retrieved cases according to the PT reported, the possibility of a misclassification bias (the wrong classification of cases, placed into the wrong categories, cytolytic or cholestatic) cannot be rules out. Considering these limitations, we are aware that the real hepatic safety profile of olaparib cannot be fully established but needs to be confirmed by the results obtained from *ad hoc* studies. On the other hand, we carried out a descriptive analysis of data derived from the EV database to identify cases of liver injury related to olaparib and to classify these cases according to their liver damage origin. A team of pharmacologists and clinicians with consolidated experience in pharmacovigilance performed this classification, with the aim to guarantee high quality standard for the overall methodological process. In addition, despite its limitations, the spontaneous reporting system represents a useful and inexpensive tool for the collection and analysis of medicine safety data for population excluded from premarketing clinical studies and for the identification of ADRs that are not detectable during the pre-marketing phase.

## 5 Conclusion

We found out that most DILI cases occurred in female patients belonging to the age group 18–64 years. DILI cases mainly were represented by cases of cytolytic origins and cases associated with liver related investigation. There were 23 fatal cases, mostly classified as “cytolytic” and “liver neoplasm or nonspecific signs”.

Additional pharmacovigilance studies assessing the hepatic safety profile of olaparib is required in light of the findings obtained from the EV. In addition, evaluation of the pathophysiological mechanism underlying the drug-induced liver damage could provide clinicians with practical resources for prevention and treatment.

## Data Availability

The raw data supporting the conclusions of this article will be made available by the authors, without undue reservation.
