# Supplementary material for: Hepatobiliary adverse drug reactions during treatment with olaparib: an analysis of data from the EudraVigilance reporting system
Source: Front Drug Saf Regul. 2026 Jan 9;5:1736759. doi: 10.3389/fdsfr.2025.1736759 (PMC12827739; doi:10.3389/fdsfr.2025.1736759)
Supplement: Supplementary file 1 [file Table1.docx]

**Supplementary Table 1.** List of PTs suggesting cytolytic and cholestatic liver damage origin.

| **Cytolytic^a^** | **Cholestatic^b^** |
| --- | --- |
| Acute hepatic failure | Cholestasis |
| Biliary fibrosis | Hyperbilirubinaemia |
| Coma hepatic | Icterus index increased |
| Hepatic atrophy | Jaundice |
| Hepatic cirrhosis | Jaundice cholestatic |
| Hepatic encephalopathy | Jaundice hepatocellular |
| Hepatic failure | Cholaemia |
| Hepatic fibrosis | Ocular icterus |
| Hepatic necrosis | Bilirubin excretion disorder |
| Hepatic steatosis | Cholestatic pruritus |
| Hepatitis fulminant | Parenteral nutrition associated liver disease |
| Hepatocellular injury | Hepatitis cholestatic |
| Hepatorenal syndrome | Reynold's syndrome |
| Non-alcoholic fatty liver | Primary biliary cholangitis |
| Portal hypertension | Immune-mediated cholangitis |
| Reye's syndrome |  |
| Hepatic cytolysis |  |
| Non-alcoholic steatohepatitis |  |
| Hepatocellular foamy cell syndrome |  |
| Subacute hepatic failure |  |
| Chronic hepatic failure |  |
| Hepatic lesion |  |
| Cryptogenic cirrhosis |  |
| Hepatic infiltration eosinophilic |  |
| Hepatic hydrothorax |  |
| Acute yellow liver atrophy |  |
| Steatohepatitis |  |
| Hepatic steato-fibrosis |  |
| Acquired hepatocerebral degeneration |  |
| Immune-mediated hepatic disorder |  |
| Autoimmune hepatitis |  |
| Chronic hepatitis |  |
| Hepatitis |  |
| Hepatitis acute |  |
| Hepatitis chronic active |  |
| Hepatitis chronic persistent |  |
| Hepatitis toxic |  |
| Ischaemic hepatitis |  |
| Radiation hepatitis |  |
| Lupus hepatitis |  |
| Allergic hepatitis |  |
| Immune-mediated hepatitis |  |
| Alloimmune hepatitis |  |

^a^ Cytolytic: ICSRs reporting at least one PT belonging to the SMQs “Drug related hepatic disorders – severe events only” (20000007) and suggesting hepatocyte cytolysis

^b^ Cholestatic: ICSRs reporting at least one PT belonging to the SMQs “Cholestasis and jaundice of hepatic origin” (20000009) and suggesting an alteration of the normal flow of bile from the liver to the intestine
